# Shifts from *cis*-to *trans*-splicing of five mitochondrial introns in *Tolypanthus maclurei*

**DOI:** 10.7717/peerj.12260

**Published:** 2021-10-01

**Authors:** Runxian Yu, Chenyu Sun, Ying Liu, Renchao Zhou

**Affiliations:** State Key Laboratory of Biocontrol and Guangdong Provincial Key Laboratory of Plant Resources, School of Life Sciences, Sun Yat-Sen University, Guangzhou, Guangdong, China

**Keywords:** Intron evolution, Mitogenome, Trans-splicing, Horizontal gene transfer, Angiosperms

## Abstract

Shifts from *cis*-to *trans*-splicing of mitochondrial introns tend to correlate with relative genome rearrangement rates during vascular plant evolution, as is particularly apparent in some lineages of gymnosperms. However, although many angiosperms have also relatively high mitogenomic rearrangement rates, very few *cis*-to *trans*-splicing shifts except for five *trans*-spliced introns shared in seed plants have been reported. In this study, we sequenced and characterized the mitogenome of *Tolypanthus maclurei*, a hemiparasitic plant from the family Loranthaceae (Santalales). The mitogenome was assembled into a circular chromosome of 256,961 bp long, relatively small compared with its relatives from Santalales. It possessed a gene content of typical angiosperm mitogenomes, including 33 protein-coding genes, three rRNA genes and ten tRNA genes. Plastid-derived DNA fragments took up 9.1% of the mitogenome. The mitogenome contained one group I intron (cox1i729) and 23 group II introns. We found shifts from *cis*-to *trans*-splicing of five additional introns in its mitogenome, of which two are specific in *T. maclurei*. Moreover, *atp1* is a chimeric gene and phylogenetic analysis indicated that a 356 bp region near the 3′ end of *atp1* of *T. maclurei* was acquired from Lamiales *via* horizontal gene transfer. Our results suggest that shifts to *trans*-splicing of mitochondrial introns may not be uncommon among angiosperms.

## Introduction

Plant mitogenomes vary markedly in size, structure and gene content among different lineages (Mower, Sloan & Alverson, 2012; Smith & Keeling, 2015), and repetitive sequences are usually the most dynamic elements in plant mitogenomes (Wynn & Christensen, 2019; Xia et al., 2020). Plant mitogenome size can increase greatly by expansion of repetitive sequences (Sloan et al., 2012; Dong et al., 2018). Meanwhile, repeat-mediated DNA rearrangement can affect plant mitogenome content and structure, and is thus considered as one of the main forces contributing to plant mitogenome evolution (Smith et al., 2010; Dong et al., 2018). One conspicuous way of repeat-mediated rearrangement for shaping mitogenomes of seed plants and some lycophytes is shifts from *cis*-to *trans*-splicing of mitochondrial introns (Chapdelaine & Bonen, 1991; Malek & Knoop, 1998; Qiu & Palmer, 2004; Hecht, Grewe & Knoop, 2011; Guo et al., 2016; Guo et al., 2020).

There are two types of plant mitochondrial introns (groups I and II), which differ in their secondary structures and splicing (Bonen, 2012). During pre-mRNA processing, group II mitochondrial introns are removed by either *cis*-or *trans*-splicing (Bonen, 2012). Non-vascular plants have no mitochondrial *trans*-splicing, while there is a number of shifts from cis-to *trans*-splicing of mitochondrial introns in seed plants and some lycophytes (Malek & Knoop, 1998; Groth-Malonek et al., 2004; Guo et al., 2016; Guo et al., 2020). A recent study found that 50–70% of mitochondrial introns in Pinaceae and cupressophyte require *trans*-splicing, indicating extensive *cis*-to *trans*-splicing shifts in these gymnosperm lineages (Guo et al., 2020).

Evolutionary shifts from *cis*-to *trans*-splicing of mitochondrial introns usually occurs *via* the DNA rearrangement-induced break in *cis*-spliced introns (Massel, Silke & Bonen, 2016). Based on available mitogenome data, shifts to *trans*-splicing in vascular plant mitogenomes tend to correlate with relative genome rearrangement rates (Hecht, Grewe & Knoop, 2011; Guo et al., 2020; Mower, 2020). On the contrary, *trans*-to *cis*-splicing shifting is expected to be unprocurable because the fortuitous rejoining of two distal intron fragments by double-strand break repair (without removing essential intron components or introducing non-essential DNA) is less likely, and no convincing cases have been found (Mower, 2020).

Among angiosperms, a total of 25 mitochondrial group II introns and one group I intron have been identified (Cho et al., 1998; Bonen, 2008; Mower, Sloan & Alverson, 2012). The group I intron cox1 i729 was acquired *via* horizontal transfer from a fungus, and further horizontal transfer events among angiosperms (Cho et al., 1998; Sanchez-Puerta et al., 2011; Sanchez-Puerta et al., 2008). Among the 25 mitochondrial group II introns, five *trans*-spliced ones (nad1i394, nad1i669, nad2i542, nad5i1455 and nad5i1477) are shared by almost all seed plants, suggesting *trans*-splicing of the five introns is the ancestral state for seed plants (Mower, 2020). Like gymnosperms, angiosperms usually have abundant repetitive sequences in their mitogenomes and relatively high rearrangement rate (Palmer & Herbon, 1988; Sloan et al., 2012; Dong et al., 2018), which may trigger *cis*-to *trans*-splicing shift of mitochondrial introns. However, *cis*-to *trans*-splicing shifts among angiosperms have been reported for only three additional mitochondrial introns, that is, cox2i373 in *Allium cepa* and *Viscum scurruloideum* (Kim & Yoon, 2010; Skippington et al., 2015), nad2i156 in *Epirixanthes elongata* (Petersen et al., 2019), and nad1i728 in diverse angiosperm lineages (Qiu & Palmer, 2004).

In this study, we assembled and characterized the mitogenome of *Tolypanthus maclurei*, a hemiparasitic plant from the family Loranthaceae (Santalales). Compared to seed plant common ancestor, shifts from *cis*-to *trans*-splicing of five additional introns were detected in this mitogenome. *Trans*-splicing of two of the five introns were only found in *T. maclurei*. To our knowledge, no any other angiosperms were reported to possess such a high number of shifts from *cis*-to *trans*-splicing of mitochondrial introns.

## Materials & methods

### Sequencing data

Illumina sequencing of an individual of *T. maclurei* was done in Yu et al. (2019). We used Trimmomatic v0.39 (Bolger, Lohse & Usadel, 2014) to filter the Illumina reads with default parameters.

### Mitogenome assembly

We used GetOrganelle v1.7.1 (Jin et al., 2020) to assemble all Illumina reads with the parameters:-F embplant_mt and-k 25, 55, 75, 95, 105, 125, and a custom mitochondrial database as the reference. The output contained 252 contigs ranging from 127 to 48,435 bp in length. We extracted the depth of coverage for each contig from the head line in the output FASTA files. Contigs with depth of coverage < 20× were discarded to exclude the potential nuclear genome sequences (Fig. S1). The remaining contigs were then searched against the custom mitochondrial database and the plastome sequence of *T. maclurei* (GenBank accession number NC_042257) by BLASTN with-evalue set to 1e−5. Only the contigs that had at least one hit > 100 bp were kept. As a result, we got 47 contigs with a total length of 344,348 bp.

To connect mitochondrial contigs and exclude plastid contigs, all Illumina reads were mapped to the 47 contigs by Bowtie2 v2.4.0 (Langmead et al., 2018) and then extracted by Samtools v1.9 (Li et al., 2009) with-F4 flag value to make a subset of Illumina reads. Then the subset was supplied to Unicycler v0.4.9 (Wick et al., 2017) to generate a graph file that was then visualized in Bandage v0.8.1 (Wick et al., 2015). We could easily identify plastid contigs and mitochondrial plastid insertions (MTPTs) because they would have much higher depth of coverage than mitochondrial contigs. After removing plastid contigs, the remaining contigs could be arranged into a circular molecule. To avoid the influence of the MTPTs on the polishing step in Unicycler, Illumina reads were remapped to the assembly using Bowtie2 and sequences of the MTPTs were carefully checked and manually curated wherever needed. All Illumina reads were then mapped to the assembly with Bowtie2, and the depth of coverage across the mitogenome was calculated in Samtools.

### Mitogenome annotation

We performed BLASTN to annotate protein-coding genes and rRNAs in the mitogenome, using mitochondrial genes from other angiosperms (Table S1) as references. The parameters used for annotation are the same as those in Skippington et al. (2017). Gene fragments and pseudogenes > 100 bp in length were also identified. Annotation of tRNAs was executed by tRNAscan-SE v2.0 (Lowe & Chan, 2016) with the “organelle” mode. MTPTs > 100 bp were identified by BLASTN with-evalue set to 1e−5 and-perc_identity set to 80, using its plastome as the reference. To show *cis*-to *trans*-splicing shifts events in the phylogeny of seed plants, mitochondrial intron information of 17 angiosperms and four gymnosperms were extracted from GenBank (Table S1). The phylogenetic tree was drawn based on the phylogenies of angiosperms shown in APG IV (2016), of gymnosperms shown in One Thousand Plant Transcriptomes Initiative (2019), and of Santalales shown in Su et al. (2015). These shift events were then labeled on the tree. To characterize the *trans*-spliced introns newly found in *T. maclurei*, we downloaded the mitogenome of *Vitis vinifera* (GenBank accession number NC_012119). BLASTN was performed to find synteny for the introns and flanking exons between *V. vinifera* and *T. maclurei* with the same parameters used to identify MTPTs.

### Identification of interspersed repeats and assessment of repeat-mediated recombination

BLASTN was used to search interspersed repeats > 50 bp in the mitogenome with the same parameters used to identify MTPTs. We assessed the recombination activity for all repeat pairs < 350 bp (the insert size of our Illumina library). For each repeat pair, we constructed two reference sequences, each with the repeat itself and 300 bp up-and downstream of the putatively repeat-mediated recombined sequences (alternative conformations). All Illumina reads were mapped to the references by Bowtie2 with the parameters: –end-to-end, –no-mixed and –no-discordant, and then the numbers of read pairs supporting alternative conformations were recorded.

### Evaluating the phylogenetic origin of mitochondrial protein-coding genes

We downloaded the sequences of mitochondrial protein-coding genes of 37 diverse angiosperms (Table S1). Additionally, mitochondrial protein-coding genes from *Erythropalum scandens* (an autotrophic species in Erythropalaceae, Santalales) and *Santalum album* (an hemiparasitic species in Santalaceae, Santalales) were extracted from their mitochondrial contigs, which were assembled based on Illumina reads sequenced by ourselves (unpublished data) and downloaded from Genbank (SRX4079976), respectively. All mitochondrial genes were aligned with MUSCLE v3.8.31 (Edgar, 2004) under the “codon” model, and the alignments were manually modified when necessary. To exclude the potential influence of RNA editing on phylogenetic analysis, all C-to-U RNA editing sites were predicted by PREP-Mt (Mower, 2005) with default parameters and then removed from the alignments. Three genes (*atp9*, *nad4L* and *rps14*) were excluded because they are too short (212, 284 and 296 bp long, respectively) after removing potential RNA editing sites. We constructed a maximum likelihood tree for each gene using RAxML (Stamatakis, 2014) with the GTR + Gamma model and 1,000 bootstrap replications. *Liriodendron tulipifer*a was used as an outgroup. For each gene, if *T. maclure*i was sister to other lineages with high bootstrap support value (BS ≥ 70%) rather than Santalales members, we would regard its origin as foreign rather than native. Also, we used GENECONV v1.81 (Sawyer, 1989) with parameters-gscale = 1 and-pairwise to evaluate if there are any chimeric genes in the *T. maclure*i mitogenome. We found that *atp1* of *T. maclurei* was a chimeric gene, with partial region showing very high identities with species of Lamiales. Further phylogenetic analyses were carried out for the identified native region and foreign region of *atp1* separately. To exclude the potential influence of substantial region length disparity (1,137 bp for the native region and 356 bp for the foreign region), we also divided the native region into three subregions (1–356, 357–712 and 713–1,137) and performed phylogenetic analyses separately. Sequence identities of the three subregions and the foreign region between *T. maclurei* and other Santalales species, and between *T. maclurei* and Lamiales species, were calculated with BLASTN. Approximately unbiased (AU) test in IQ-TREE v1.6 (Nguyen et al., 2015) was performed to verify the origin of partial sequence of *atp1 via* horizontal gene transfer (HGT). We tested two constrained topologies: *T. maclurei* and *Dendrophthoe pentandra* clustered with *E. scandens* and *Malania oleifera* (constraint #1), and *T. maclurei* and *D. pentandra* clustered with *S. album* and *Loranthus europaeus* (constraint #2). *P*-values of the AU tests were calculated under 10,000 RELL replicates.

## Results & discussion

### Mitogenome structure and gene content

**Figure 1:**
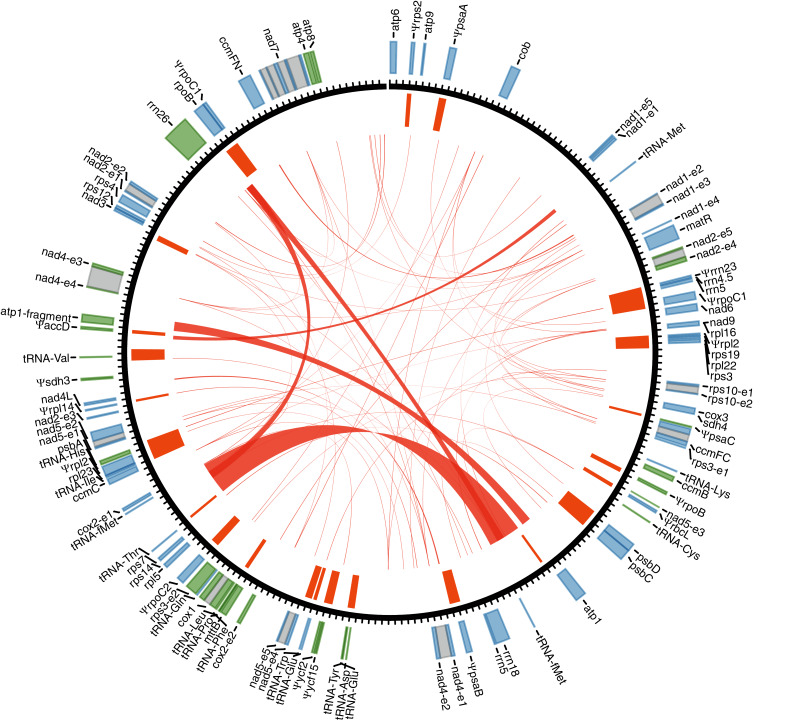
Gene annotation for the mitogenome of *Tolypanthus maclurei*. Gene annotation is shown in the outer track. Genes colored as blue and green are transcribed clockwise and counter-clockwise, respectively. Grey boxes represent putative *cis*-spliced introns. The unit length of the tick marks is one kb. Mitochondrial plastid insertions (MTPTs) are shown as orange bars in the inner tracks, with the width corresponding to the size of MTPTs. Repeat pairs > 50 bp are indicated by red links.

With Illumina sequencing data, we assembled a circular 256,961-bp mitogenome for *Tolypanthus maclurei* (Fig. 1). Relative to other species in Santalales with available sequenced mitogenome, the mitogenome size of *T. maclurei* is the second smallest, higher than that of *Viscum scurruloideum* (Skippington et al., 2015), but much smaller than those of *Ombrophytum subterraneum* (Roulet et al., 2020) and *Lophophytum mirabile* (Sanchez-Puerta et al., 2019). The overall GC content is 44.4%, which is similar to other species of Santalales (44.2–47.4%) (Skippington et al., 2015; Sanchez-Puerta et al., 2019; Roulet et al., 2020). Depth of coverage across the whole mitogenome except the MTPTs are relatively even, indicating the continuity of our assembly (Fig. S2). There were 74 repeat pairs with length > 50 bp, totally occupying 9.61% (24,681 bp) of the mitogenome. Among these repeat pairs < 350 bp in length, only eleven of them (ranging from 51 to 211 bp) were found to mediate recombination (Table S2) and the numbers of read pairs supporting rearranged conformations were relatively low (ranging from 1 to 26), indicating limited recombination activity for these relatively short repeats in this mitogenome.

We identified 46 mitochondrial genes in the *T. maclurei* mitogenome, including 33 protein-coding genes, three rRNA genes and 10 tRNA genes (not including genes from MTPTs). Among the protein-coding genes, all 24 “core genes” which are usually present in seed plants (Mower, Sloan & Alverson, 2012) as well as eight ribosomal protein genes and one succinate dehydrogenase subunit gene (*sdh4*) which are variably present in seed plants were annotated. *sdh3* was pseudogenized due to substantial truncation in the 3’ end. The gene regions (not including introns) represented 14.2% of the mitogenome. Three of the four conserved gene clusters are present in the mitogenome, namely, *cox3*-*sdh4*, *18S*-*5S rRNAs* and *nad3*-*rps12* (Gagliardi & Binder, 2007; Richardson et al., 2013), and *rps19*-*rps3*-*rpl16* gene cluster was destroyed due to the loss of *rps19* and the shift from *cis*-to *trans*-splicing of the intron rps3i74 (see details latter).

By searching the *T. maclurei* mitogenome against its own plastome, 31 regions were found to be highly similar to the sequence of the latter (MTPTs) (Table S3). The lengths of these regions ranged from 106 to 2,617 bp with 94.7% to 100.0% identity to the counterparts in its plastome, accounting for 9.1% of the mitogenome. These MTPTs contain 20 protein-coding genes, three rRNA genes and eight tRNA genes, among which 15 protein-coding genes and the *rrn23* gene were pseudogenized because of substantial truncation or frameshift mutations. The presence of nonfunctional plastid genes (pseudogenes) is a common feature in plant mitogenomes (Mower, Sloan & Alverson, 2012).

### Mitochondrial introns

**Figure 2:**
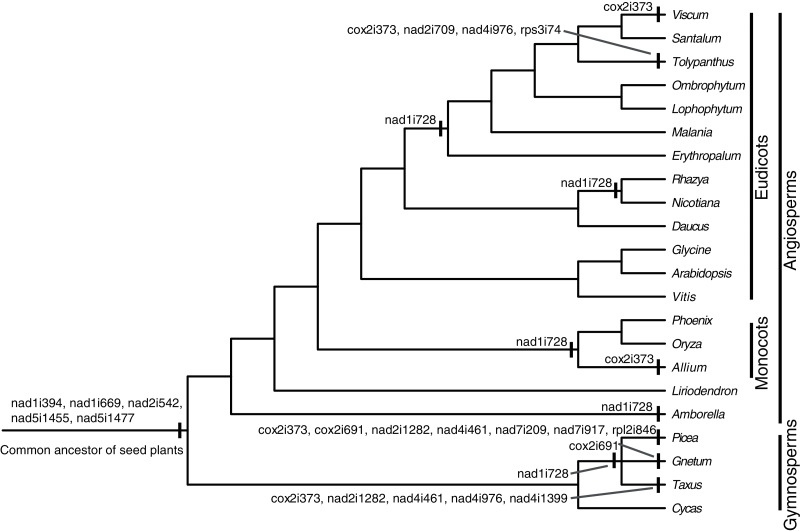
*Cis*-to-*trans* shift events of mitochondrial introns in *Tolypanthus maclurei* and other representative seed plants. The phylogenetic tree was drawn based on the phylogenies of angiosperms shown in APG IV (2016), of gymnosperms shown in One Thousand Plant Transcriptomes Initiative (2019), and of Santalales shown in Su et al. (2015). Shift events were labeled on the tree.

There were 24 introns in the *T. maclurei* mitogenome, including one group I intron (cox1i729) and 23 group II introns. A total of 13 of the 23 group II introns were cis-spliced ones, and the remaining ten were considered as trans-spliced ones. Nine introns were identified as *trans*-spliced based on discontinuity of adjacent exons (Fig. 2). The one exception was nad1i394 whose flanking exons (exon1 and exon2) were adjacent to each other in the same orientation. We still considered it as a *trans*-spliced intron, in that it is *trans*-spliced in all other seed plants and that shift from trans-to cis-splicing seems unlikely (Mower, 2020). Among the ten *trans*-spliced introns, five (nad1i394, nad1i669, nad2i542, nad5i1455 and nad5i1477) are *trans*-spliced in the common ancestor of seed plants (Bonen, 2008; Mower, Sloan & Alverson, 2012; Guo et al., 2020), and the remaining five (cox2i373, nad1i728, nad2i709, nad4i976 and rps3i74) is the outcome of shifts from *cis*-spliced introns. *Trans*-splicing of nad1i728 have been found in many lineages of seed plants (Fig. 2), such as *Taxus baccata* from gymnosperms (Guo et al., 2020), and *Oryza sativa* and *Nicotiana tabacum* from angiosperms (Notsu et al., 2002; Qiu & Palmer, 2004; Sugiyama et al., 2005). In Santalales, *trans*-splicing of nad1i728 appears to be a synapomorphy because it is observed in all species of Santalales with available information (Fig. 2). Shift from *cis*-to *trans*-splicing in cox2i373 was relatively rare, only previously reported in two gymnosperms, *Picea abies* and *Ta. baccata* (Guo et al., 2020), and two angiosperms, *Allium cepa* (Kim & Yoon, 2010) and *V*. *scurruloideum* (Skippington et al., 2015). Based on phylogenetic positions of *V. scurruloideum* and *T. maclurei* and intron type for the species in Santalales, shifts to *trans*-splicing of cox2i373 in *V. scurruloideum* and *T. maclurei* should be independently evolved. Likewise, shifts to *trans*-splicing of nad4i976 in *Ta. baccata* and *T. maclurei* must be independently evolved as well. While shifts to *trans*-splicing for each of the three introns evolved at least twice in seed plants, to our knowledge, those of the remaining two introns, nad2i709 and rps3i74, are confined to *T. maclurei* only. Lineage-specific shifts to *trans*-splicing for mitochondrial introns were also be found in other plants, such as nad2i156 in *Epirixanthes elongata* (Petersen et al., 2019), suggesting that shifts to *trans*-splicing of additional mitochondrial introns in angiosperms might not be uncommon.

**Figure 3:**
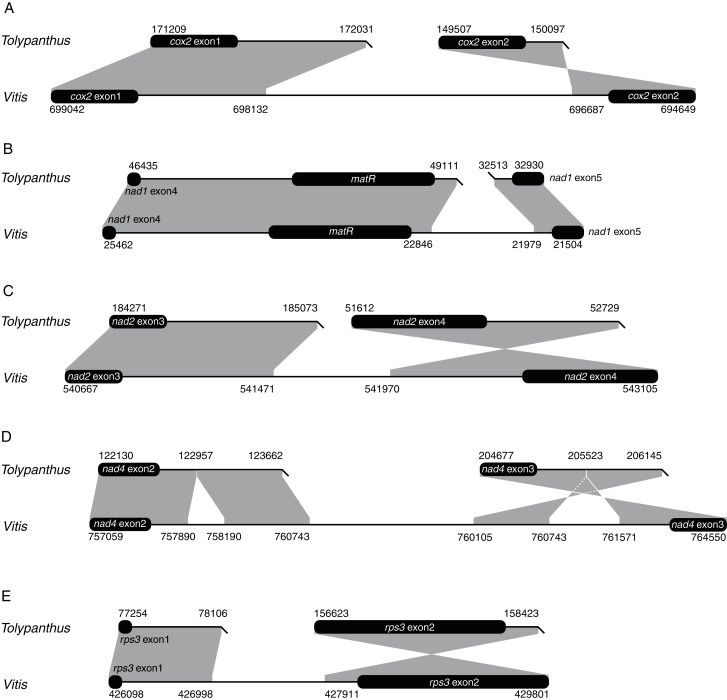
Schematic diagrams of synteny analysis of five mitochondrial introns between *Tolypanthus maclurei* and *Vitis vinifera*. Flanking exons are shown as black rounded rectangles. Gray shading with genomic coordinates indicates homologous regions between mitogenomes of the two species. (A) cox2i373. (B) nad1i728. (C) nad2i709. (D) nad4i976. (E) rps3i74.

We also explored how shifts from *cis*-to *trans*-splicing of the five introns occurred by comparing the five introns of *T. maclurei* with those of *Vitis vinifera*. Like those in *Liriodendron tulipifera*, the five introns in *Vitis vinifera* are all *cis*-spliced ones (Goremykin et al., 2008; Richardson et al., 2013), representing the ancestral states of these mitochondrial introns. For each of the five introns in *T. maclurei*, high sequence identity (>80%) between the two intron ends of *V. vinifera* and their counterparts in *T. maclurei* was detected (Fig. 3). The intron nad1i728 of *T. maclurei* was broken downstream of *matR*, leading to the separation of two fragments 14 kb away from each other. Compared with *cis*-spliced nad1i728 in *V. vinifera*, an 866 bp region was lost in *T. maclurei*. Similar situation was found in this intron of *P. abies* (Guo et al., 2020). Likewise, other four introns also lost sequence synteny in the middle of these introns between *T. maclurei* and *V. vinifera* because of intron break in *T. maclurei*. Moreover, the two exons originally flanking these introns were no longer in the same orientation (Fig. 3).

Repeat-mediated recombination is prevalent in angiosperm mitogenomes, leading to coexistence of different genome conformations (Palme & Shields, 1984; Alverson et al., 2011; Dong et al., 2018). If recombination occurs in the middle of *cis*-spliced introns, *cis*-spliced introns may shift to trans-spliced ones (Guo et al., 2020; Mower, 2020). However, we don’t observe any repeats that likely contributed to these shifts around the intron breakpoints in the *T. maclurei* mitogenome. Sequences of the repeat pairs might be translocated after repeated recombination and/or too divergent to be no longer recognized as repeats. Also, other types of DNA rearrangement may result in these shifts.

### Phylogenetic origins of mitochondrial protein-coding genes

**Figure 4:**
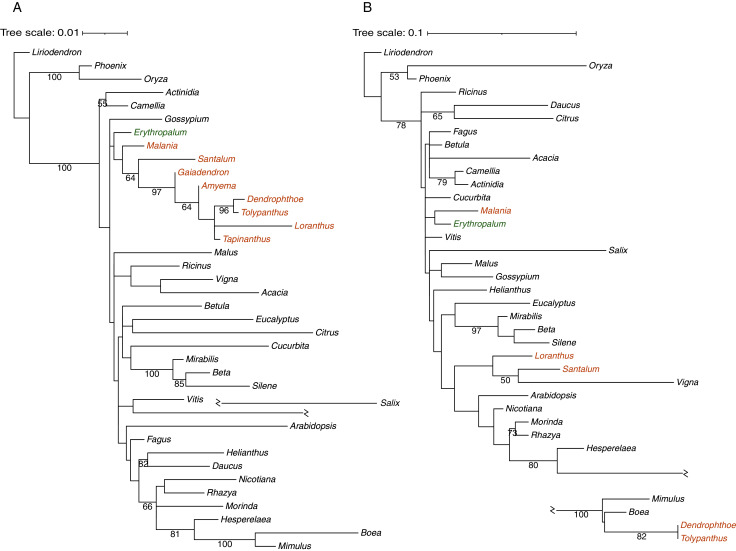
Phylogenetic analyses of *Tolypanthus maclurei* and other 37 angiosperms based on sequences of two regions of mitochondrial *atp1*. (A) Maximum likelihood tree for region I. (B) Maximum likelihood tree for region II. *Gaiadendron*, *Amyema* and *Tapinanthus* were excluded because their region II of *atp1* are too short (<50 bp) with available sequences. Bootstrap support values ≥ 50% are shown above the branches. Hemiparasitic and autotrophic species in Santalales are shown in orange and green, respectively.

Phylogenetic analysis was carried out to infer the origins of 30 mitochondrial protein-coding genes in *T. maclurei* (Fig. S3). Our gene conversion analyses found that *atp1* of *T. maclurei* was a chimeric gene: its region I (1,137 bp, nucleotide positions 1–1,137) and region II (356 bp, nucleotide positions 1,138–1,493) exhibit high sequence identities with species of Santalales and Lamiales, respectively. Region III (13 bp, nucleotide positions 1,494–1,506) is too short and divergent to infer its origin. Separate phylogenetic analyses for regions I and II indicated that region I was native (Fig. 4A), while region II was acquired from Lamiales, both with high bootstrap support (Fig. 4B). Further separate phylogenetic analyses for the three subregions of region I, which were divided into identical or similar length to region II, also supported the native nature of region I of *T. maclurei* (Fig. S4). This was also supported by sequence identity analysis. Sequence identities of the three subregions between *T. maclurei* and other Santalales species (95.2–99.5%, 96.1–100% and 98.5–100% for the three subregions, respectively) were higher than those between *T. maclurei* and Lamiales species (94.1–94.4%, 93.7–95.7% and 94.6–95.4%), while an opposite trend was observed for region II (88.9–91.6% between *T. maclurei* and other Santalales species *versus* 95.2–96.0% between *T. maclurei* and Lamiales species). The AU tests for region II with constrained topologies also rejected vertical inheritance of *atp1* (*P* < 0.01 for both constraints). Notably, *atp1* of *Dendrophthoe pentandra* (another hemi-parasite from Loranthaceae) shares this chimeric nature, indicating the occurrence of HGT and gene conversion in their common ancestor. For the other 29 genes, *T. maclurei* either clustered with other Santalales species with mostly high and modest bootstrap supports (BS > 50%), or grouped with non-Santalales species but with very low bootstrap supports (BS < 50%). However, Santalales species fail to form a well-supported cluster for almost all of these genes, so our data cannot infer their origins.

Parasitic plants evolved haustoria, which is used to invade the host and absorb water and nutrients, building a bridge for genetic material movement between parasitic plants and their hosts. This intimate association can contribute to host-to-parasite HGT, as shown in some parasitic plants (Davis & Xi, 2015; Sanchez-Puerta et al., 2019; Yang et al., 2019; Roulet et al., 2020; Cai et al., 2021). Mitogenomes of parasitic plants such as *Cynomorium coccineum*, *Rafflesia* spp., *Lophophytum mirabile* and *Ombrophytum subterraneum*, harbor extensive mitogenome fragments from their hosts (Xi et al., 2013; Cusimano & Renner, 2019; Sanchez-Puerta et al., 2019; Roulet et al., 2020). The hemiparasitic plant *T. maclurei* parasitizes many host plants, including *Eriobotrya japonica*, *Vernicia fordii*, and *Camellia* spp. (Qiu & Gilber, 2003), and therefore, HGT of mitochondrial gene(s) in this species is not surprising. Similar to the case in *T. maclurei*, *atp1* of a holoparasitic plant, *Pilostyles thurberi*, is also a chimeric gene and shows HGT of its partial sequence from its host (Barkman et al., 2007). This gene also shows parasite-to-host transfer in *Plantago* (Mower et al., 2010).

## Conclusions

In this study, we reported the first mitogenome in the family Loranthaceae. The mitogenome size of *Tolypanthus maclurei* is 256,961 bp, relatively small in Santatales. It possesses a normal gene content and massive MTPTs. Compared with seed plant common ancestor, we found the shifts from *cis*-to *trans*-splicing of five additional mitochondrial introns in *T. maclurei*, and two of them are specific in *T. maclurei*. Phylogenetic analysis showed that a 356 bp region near the 3′ end of *atp1* of *T. maclurei* was acquired from Lamiales *via* HGT. Our study suggests that shifts from *cis*-splicing to *trans*-splicing of mitochondrial introns might not be uncommon in angiosperms.

## Supplemental Information

10.7717/peerj.12260/supp-1 Supplemental Information 1 A scatter diagram showing depth of coverage and length of the assembled contigs of *Tolypanthus maclurei* based on the GetOrganelle output.

10.7717/peerj.12260/supp-2 Supplemental Information 2 Depth of coverage across the *Tolypanthus maclurei* mitogenome based on Illumina reads.

10.7717/peerj.12260/supp-3 Supplemental Information 3 Maximum likelihood tree of 40 angiosperms based on sequences of 29 mitochondrial protein-coding genes. Bootstrap support values ≥ 50% are shown above the branches. Hemiparasitic and autotrophic species in Santalales are shown in orange and green, respectively.

10.7717/peerj.12260/supp-4 Supplemental Information 4 Phylogenetic analyses of *Tolypanthus maclurei* and other 37 angiosperms based on sequences of there subregions of region I of mitochondrial *atp1*. Bootstrap support values ≥ 50% are shown above the branches. Hemiparasitic and autotrophic species in Santalales are shown in orange and green, respectively.

10.7717/peerj.12260/supp-5 Supplemental Information 5 Mitochondrial sequence data used in this study.

10.7717/peerj.12260/supp-6 Supplemental Information 6 Repeat content and evaluation of repeat-mediated recombination in the mitogenome of *Tolypanthus maclurei*.

10.7717/peerj.12260/supp-7 Supplemental Information 7 Mitochondrial plastid insertions (MTPTs) in the *Tolypanthus maclurei* mitogenome.

## Additional Information and Declarations

### DNA Deposition

The following information was supplied regarding the deposition of DNA sequences: The mitogenome of *Tolypanthus maclurei* is available at GenBank: MZ343374. The mitochondrial protein-coding genes of *Erythropalum scandens* are available at GenBank: MT888102 to MT888134.

### Data Availability

The following information was supplied regarding data availability: The DNA-seq reads are available at GenBank: PRJNA735335.
