# Supplementary figures and images for: Shifts from cis-to trans-splicing of five mitochondrial introns in Tolypanthus maclurei
Source: PeerJ. 2021 Oct 1;9:e12260. doi: 10.7717/peerj.12260 (PMC8489412; doi:10.7717/peerj.12260)

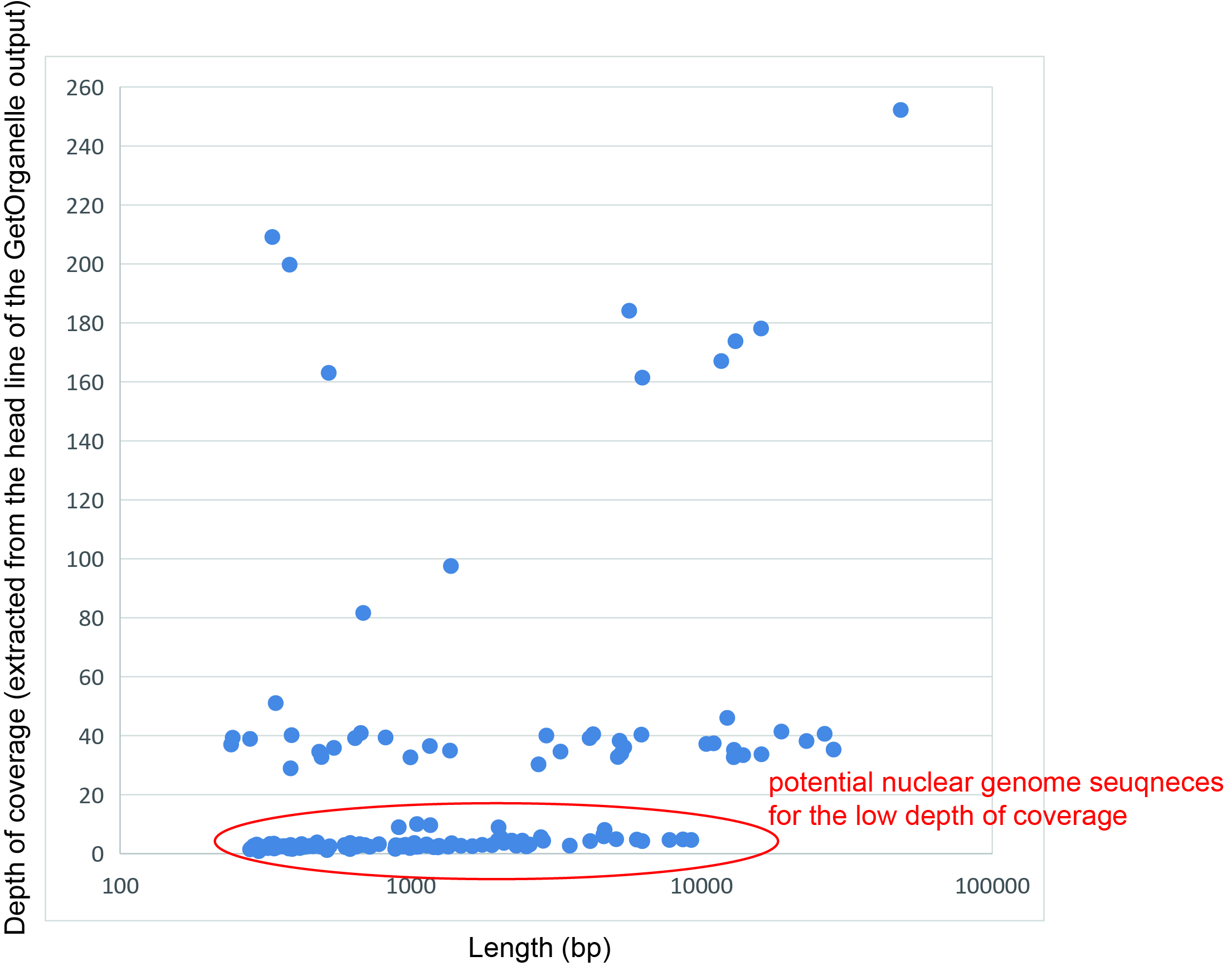

Supplement: Supplemental Information 1 [file peerj-09-12260-s001.jpg]

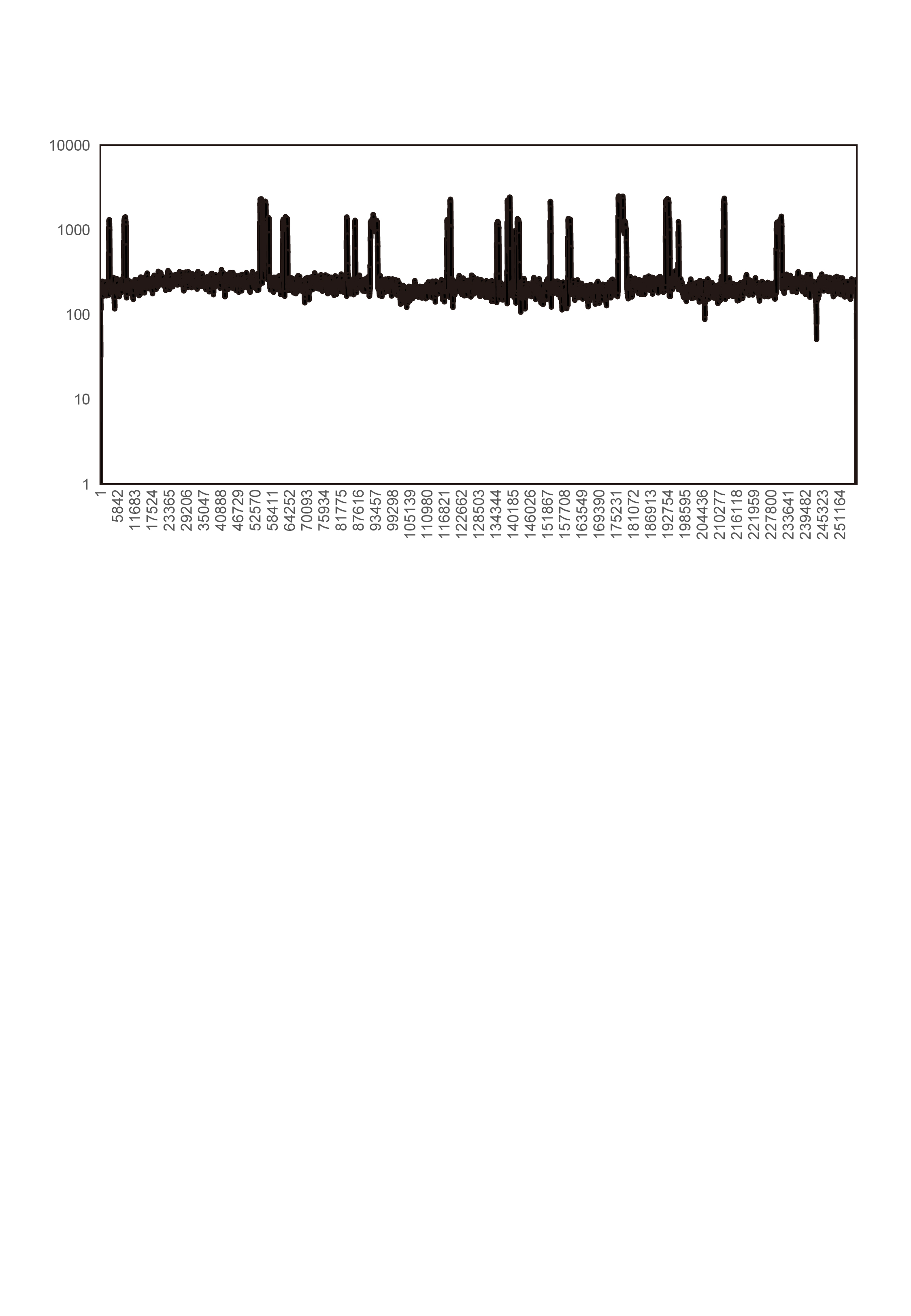

Supplement: Supplemental Information 2 [file peerj-09-12260-s002.jpg]

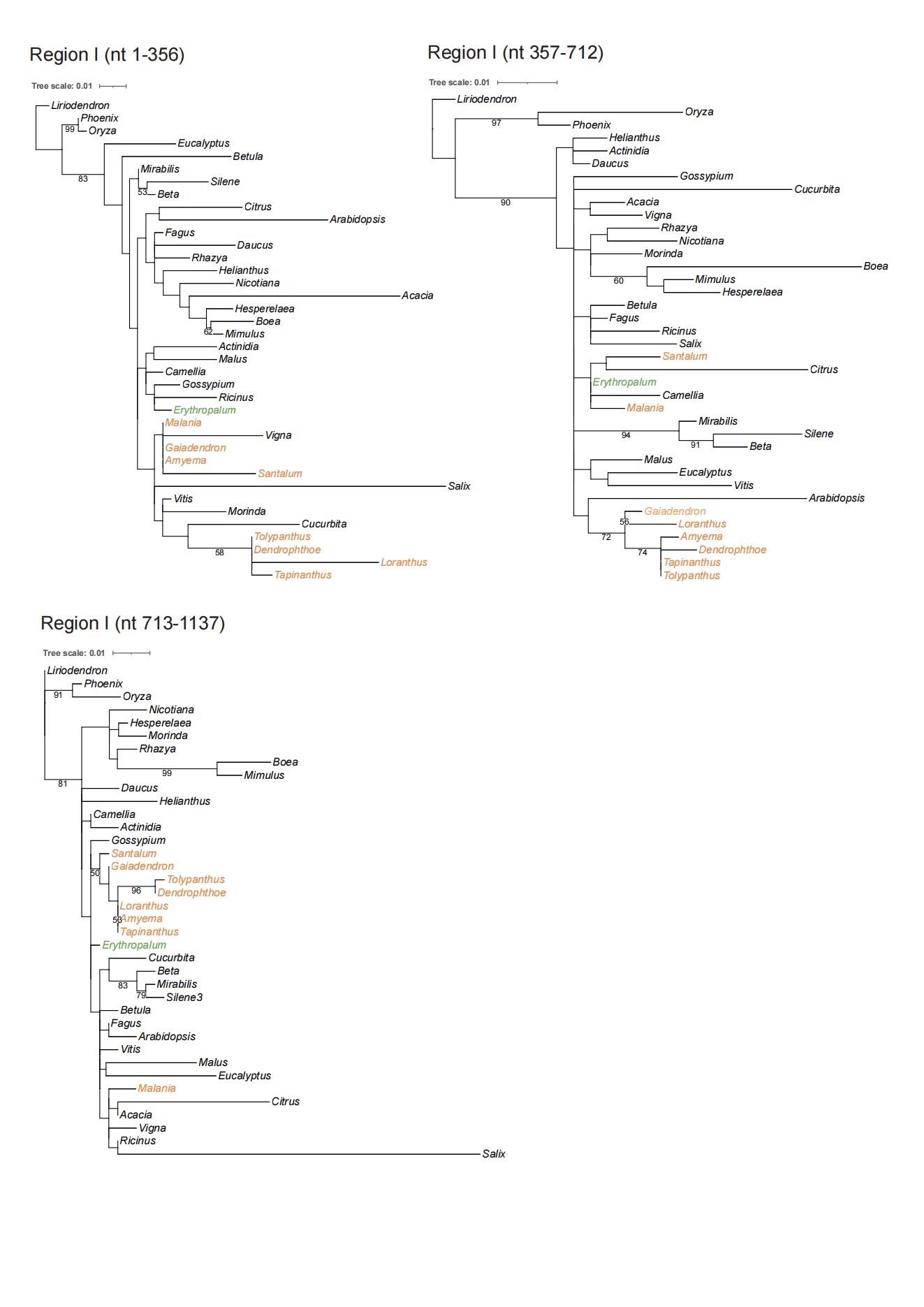

Supplement: Supplemental Information 4 — Bootstrap support values ≥ 50% are shown above the branches. Hemiparasitic and autotrophic species in Santalales are shown in orange and green, respectively. [file peerj-09-12260-s004.jpg]
